# Supplementary material for: Hamsters with long COVID present distinct transcriptomic profiles associated with neurodegenerative processes in brainstem
Source: Nat Commun. 2025 Jul 22;16:6714. doi: 10.1038/s41467-025-62048-7 (PMC12283958; doi:10.1038/s41467-025-62048-7)
Supplement: Supplementary file 1 — Supplementary information [file 41467_2025_62048_MOESM1_ESM.pdf]

Supplementary information for

Hamsters with long COVID present distinct transcriptomic profiles associated with neurodegenerative processes in brainstem

Anthony Coleon[1], Florence Larrous[1], Lauriane Kergoat[1], Magali Tichit[2], David Hardy[2], Thomas Obadia[3,4], Etienne Kornobis[3,5], Hervé Bourhy[1] and Guilherme Dias de Melo[1]*

[1] Institut Pasteur, Université Paris Cité, Lyssavirus Epidemiology and Neuropathology Unit, F-75015 Paris, France

[2] Institut Pasteur, Université Paris Cité, Histopathology Core Facility, F-75015 Paris, France

[3] Institut Pasteur, Université Paris Cité, Bioinformatics and Biostatistics Hub, F-75015 Paris, France

[4] Institut Pasteur, Université Paris Cité, G5 Infectious Diseases Epidemiology and Analytics, Paris, France.

[5] Institut Pasteur, Université Paris Cité, Plate-forme Technologique Biomics, F-75015 Paris, France

*Corresponding author:
Guilherme Dias de Melo
Lyssavirus Epidemiology and Neuropathology Unit
Institut Pasteur
25-28, rue du Dr. Roux, 75724 Paris Cedex 15, France
Tel: +33 1 44 38 88 49
e-mail: guilherme.dias-de-melo@pasteur.fr

Supplementary Figure 1

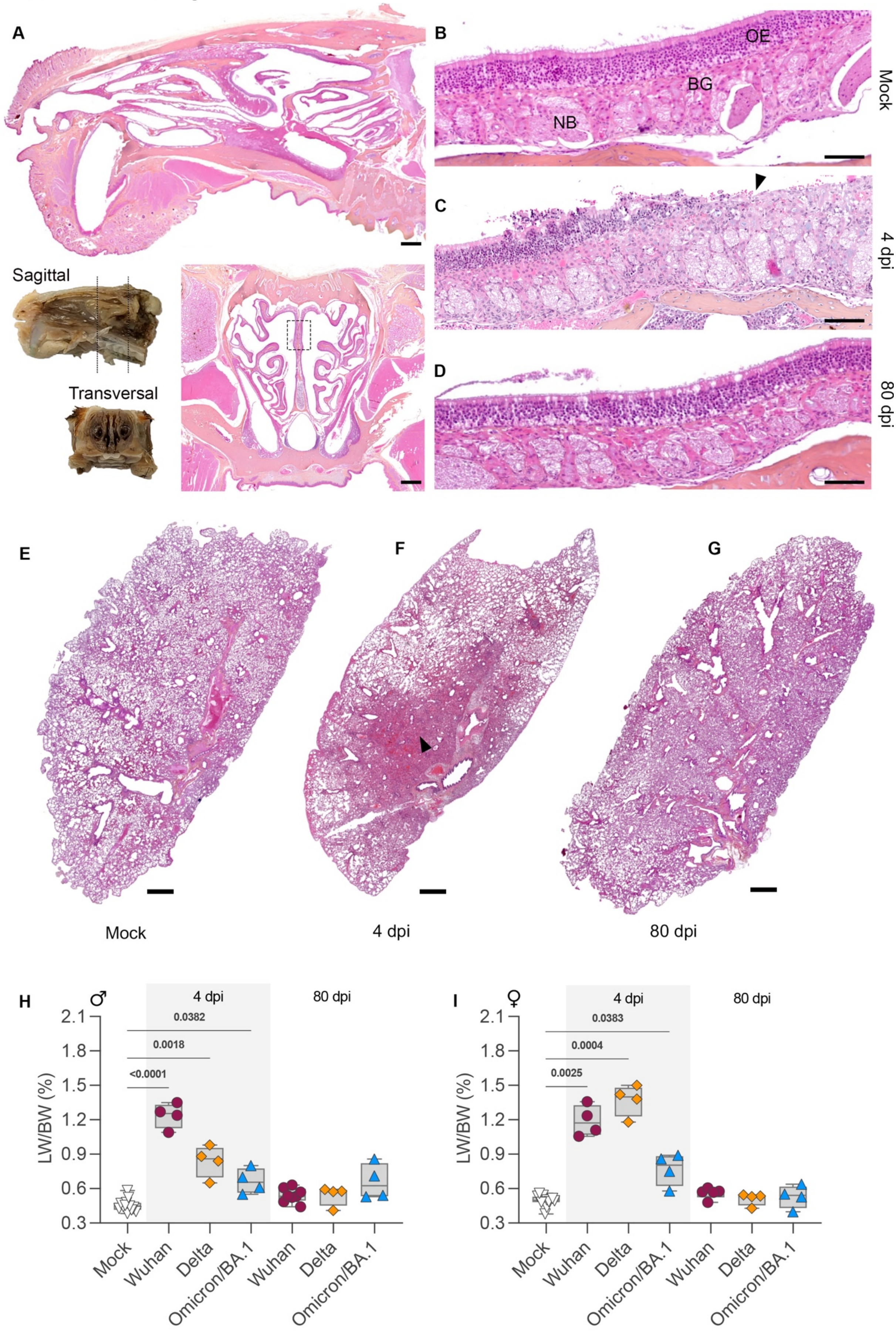

Supplementary Fig. 1. Histopathological analyses in the airways of hamsters intranasally-inoculated with SARS-CoV-2 Wuhan. (A) Formalin-fixed and decalcified macroscopic and submacroscopic views of the nasal cavity of a hamster after a sagittal (upper panels) or a transversal section (bottom panels). The dotted lines in the sagittal view indicate the sections to obtain the transversal view. The dotted square in the submacroscopic transversal view (septum) indicates the region from where the images B-D were obtained. (B-D) Olfactory mucosa from a mock-infected hamster (B), and infected hamsters at 4 days post-infection (dpi; C) and 80 dpi (D). OE: olfactory epithelium, BG: Bowman's gland, NB: nerve bundles (*filia olfactoria*). Note the extensive destruction of the olfactory epithelium (arrowhead) at 4 dpi. (E-G) Submacroscopic views of the lung of a mock-infected hamster (E), and infected hamsters at 4 dpi (F) and 80 dpi (G). Note an extensive zone of inflammation, congestion and necrosis (arrowhead) at 4 dpi. Representative images: mock-infected (n=4 males + 4 females), 4 dpi (n=4 males + 4 females), 80 dpi (n=4 males + 4 females). Scale bars: A macroscopic views = 5 mm; sub-macroscopic views = 1 mm, B-D = 100 µm, E-G = 1 mm. (H, I) Lung weight-to-body weight (LW/BW) ratio measured at 4 dpi and 80 dpi in male (H) and female (I) hamsters. Horizontal lines indicate the median and the interquartile range. Kruskal-Wallis test followed by the Dunn's multiple comparisons test (the adjusted p value is shown if $p < 0.05$). Related to Fig. 1-2.

Supplementary Figure 2

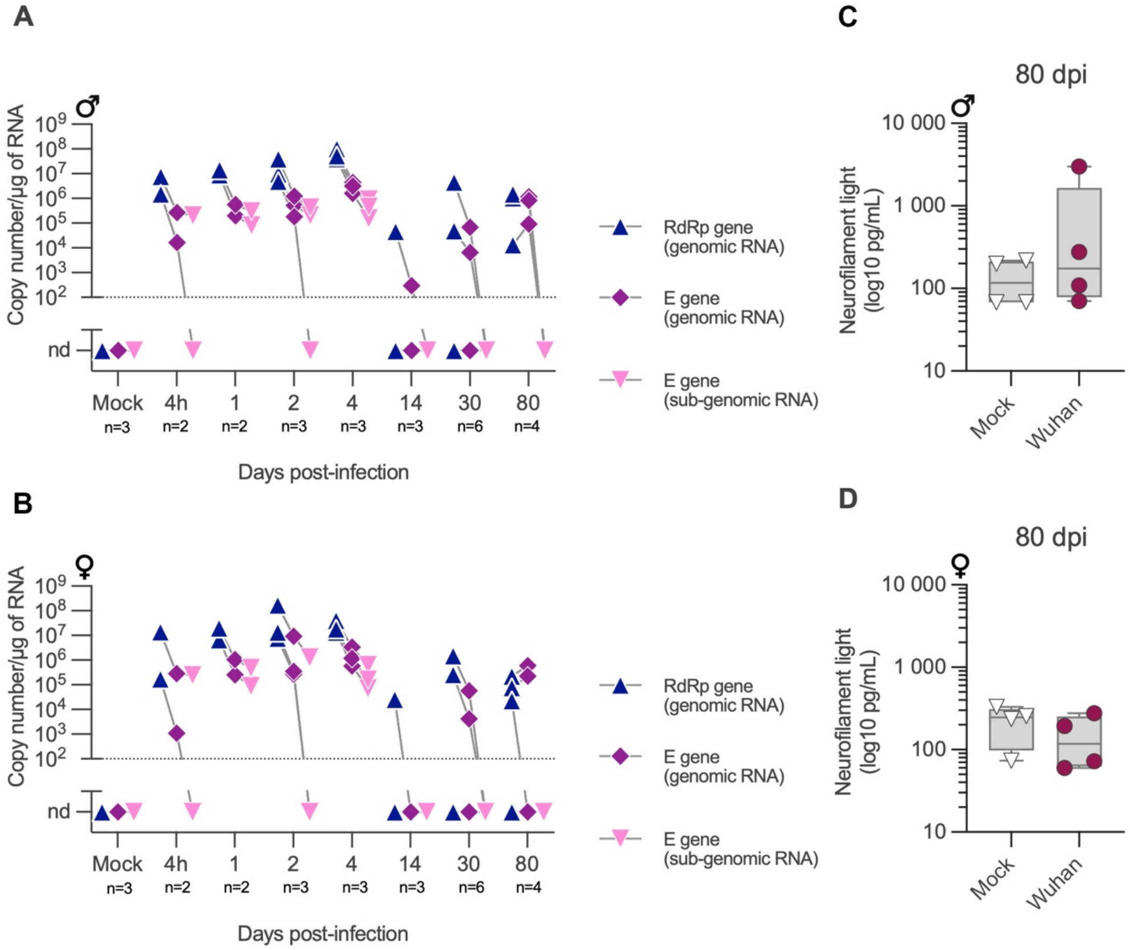

Supplementary Fig. 2. Viral load in the brainstem and serum neurofilament light chain levels of hamsters infected with SARS-CoV-2 Wuhan. (A-B) Viral RNA load kinetics in the brainstem of male (A) and female (B) hamsters infected with Wuhan evaluated at 4 hours, 1, 2, 4, 14, 30 and 80 dpi. Genomic and sub-genomic viral RNA were assessed based on the RdRp and E gene sequence. Each symbol represents one animal. Gray lines connect symbols from the same animals. Mock, 2dpi, 4dpi, 14 dpi and 30 dpi (n=3/group): 4h, 1dpi and 14dpi (n=2/group); 80dpi (n=4/group). (CD) Comparison of serum neurofilament light chain (NfL) levels between mock and Wuhan-infected male (C) and female (D) hamsters at 80 days post infection (dpi). Box and whisker plots (median, first and third quartiles, minimum and maximum). Individual values are also shown (n=4/group). Related to Fig.1.

Supplementary Figure 3

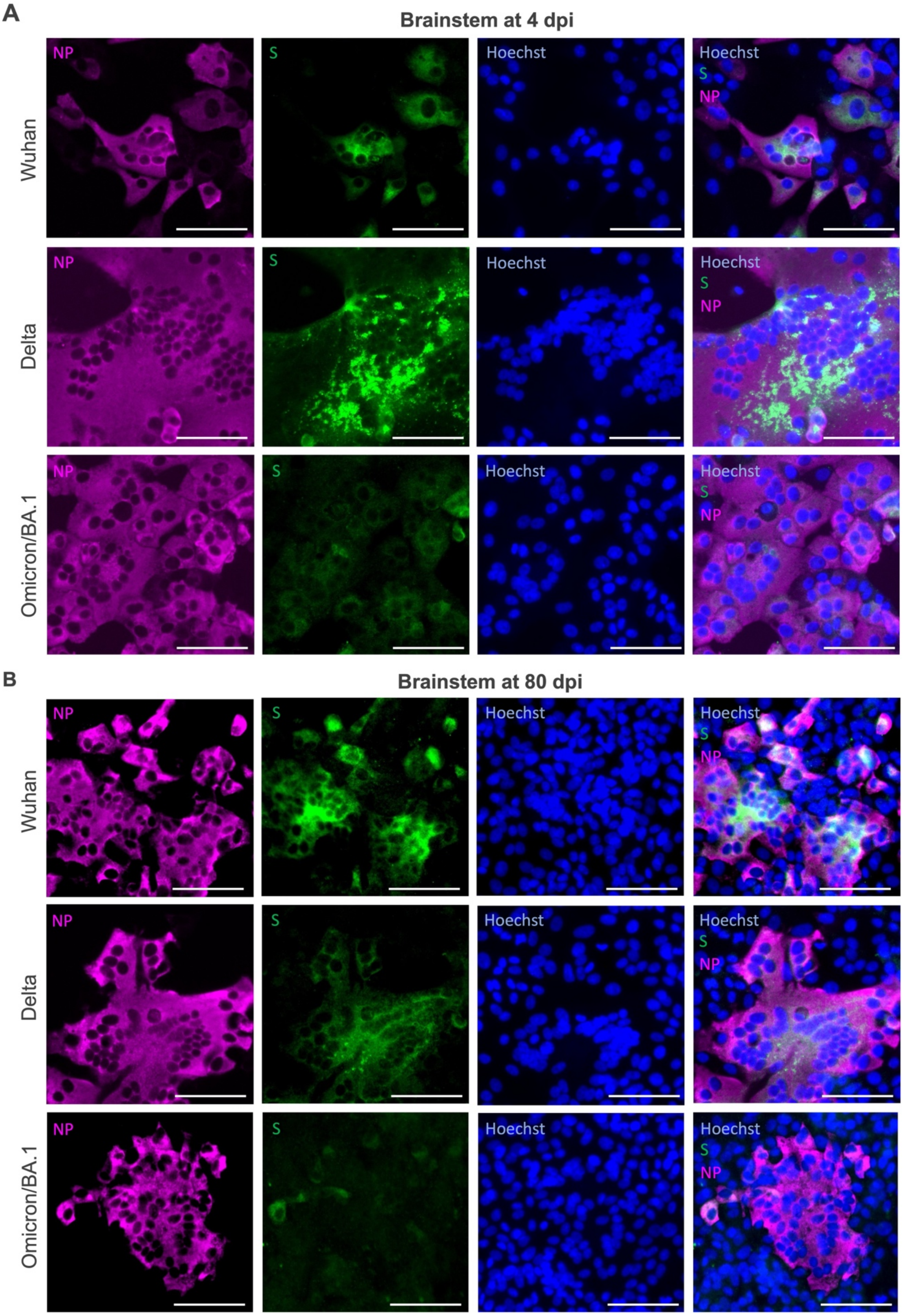

Supplementary Fig. 3. Imaging of SARS-CoV-2 isolation from the brainstem of hamsters infected with Wuhan or the variants Delta and Omicron/BA.1. (A-B). Immunofluorescence imaging of SARS-CoV-2 isolation from the brainstem of hamsters at 4 dpi (A) and 80 dpi (B). Labeling for the SARS-CoV-2 nucleoprotein (NP, magenta) and spike (S, green). The nuclei of Vero-E6 cells are stained with Hoechst (blue). Scale bar = 75 µm. Related to Fig.1.

Supplementary Figure 4

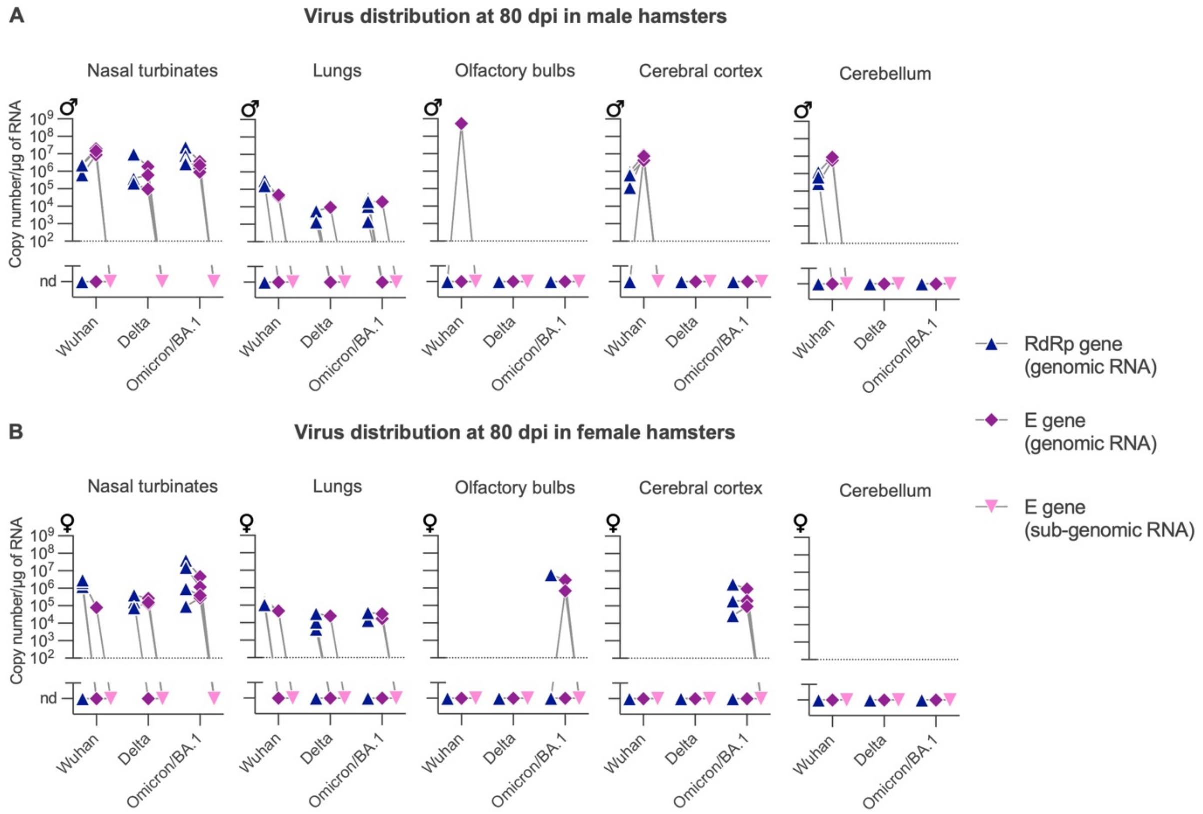

Supplementary Fig. 4. SARS-CoV-2 distribution in different tissues of hamsters infected with Wuhan or the variants Delta and Omicron/BA.1 at 80 days post infection (dpi). (A-B) Virus distribution at 80 dpi in different tissues: nasal turbinates, lungs, olfactory bulbs, cerebral cortex and cerebellum infected with SARS-CoV-2 Wuhan, Delta or Omicron/BA.1 in male (A) or female (B) hamsters (n=4/group). Genomic and sub-genomic viral RNA were assessed based on the RdRp and E gene sequence. Each symbol represents one animal. Gray lines connect symbols from the same animals. Related to Fig.1.

Supplementary Figure 5

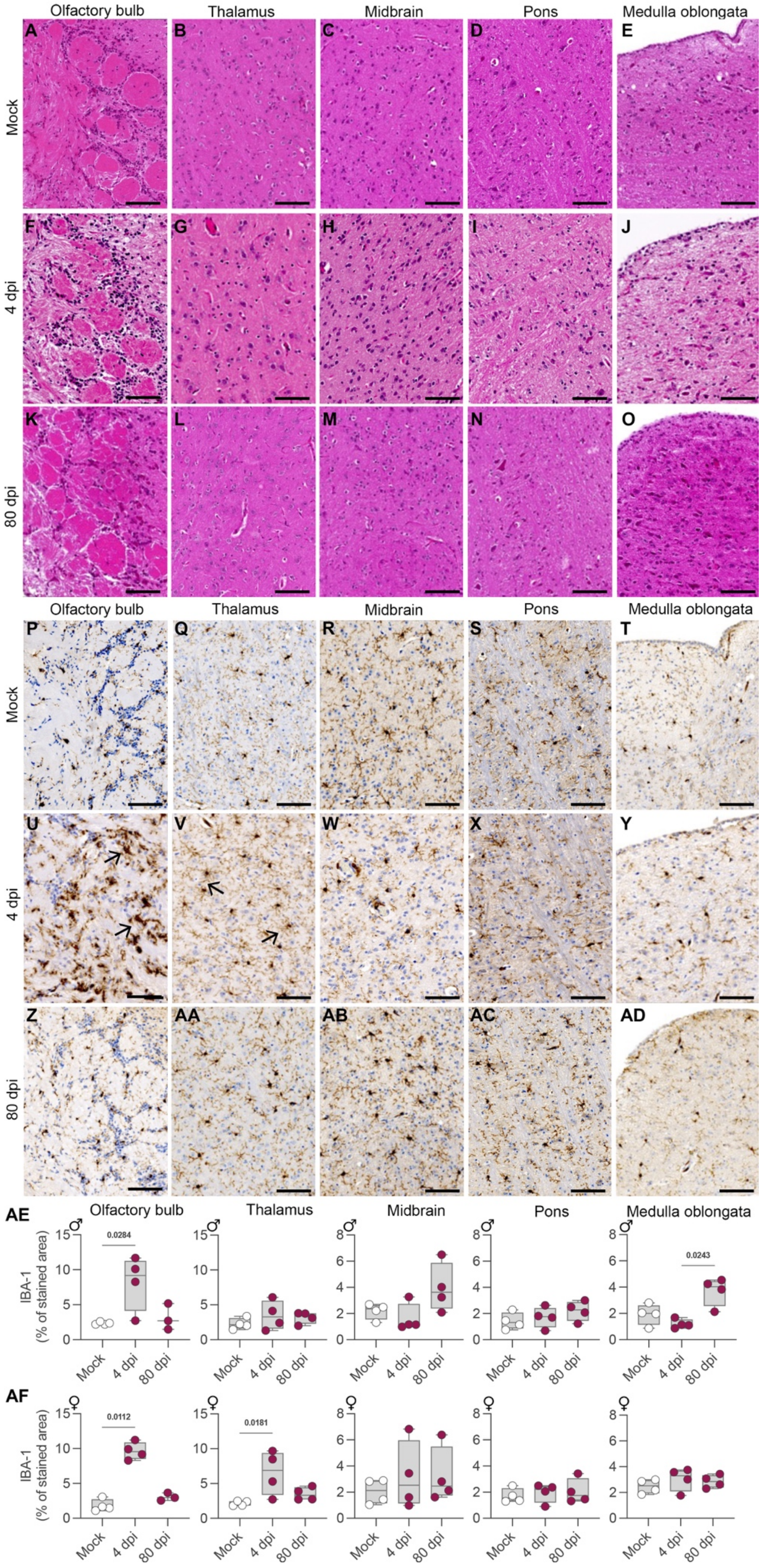

Supplementary Fig. 5. Histopathological analysis in the brain of hamsters intranasally-inoculated with SARS-CoV-2 Wuhan. (A-E) HE staining of the brain of a mock-infected hamster: olfactory bulb (A), thalamus (B), midbrain (C), pons (D) and medulla oblongata (subependymal zone; E). (F-J) HE staining of the brain at 4 days post-infection (dpi). (K-O) HE staining of the brain at 80 dpi. No major alteration was observed. Scale bars = 100 μm. Representative images of hematoxylin-eosin staining. (P-AD) Microglial detection (IBA-1+) in the brain of hamsters. (P-T) Iba-1 staining in the brain of mock-infected hamsters. (I-M) Microglial detection in the brain of a hamster at 4 dpi, where an intense microglial reactivity (hyperplasia with thicker cell processes) can be observed in the olfactory bulbs (U, arrow) and discrete microgliosis can be randomly found in the thalamus (V, arrow). (Z-AD) At 80 dpi, microglial cells were in general not reactive. Scale bars = 100 μm. Representative images of immuno-histochemistry to detect microglia using IBA-1 as marker. Scale bars = 100 μm. (AE-AF) Quantification of the percentage of the IBA-1 stained area in the olfactory bulb, thalamus, midbrain, pons, and medulla oblongata of male (S) and female (T) hamsters. Mock-infected (n=4 males + 4 females), hamsters infected at 4 dpi (n=4 males + 4 females), and at 80 dpi (n=4 males + 4 females). Box and whisker plots (median, first and third quartiles, minimum and maximum). Individual values are also shown. Kruskal-Wallis test followed by the Dunn's multiple comparisons test (the adjusted p value is indicated if $p < 0.05$). Related to Fig. 2.

Supplementary Figure 6

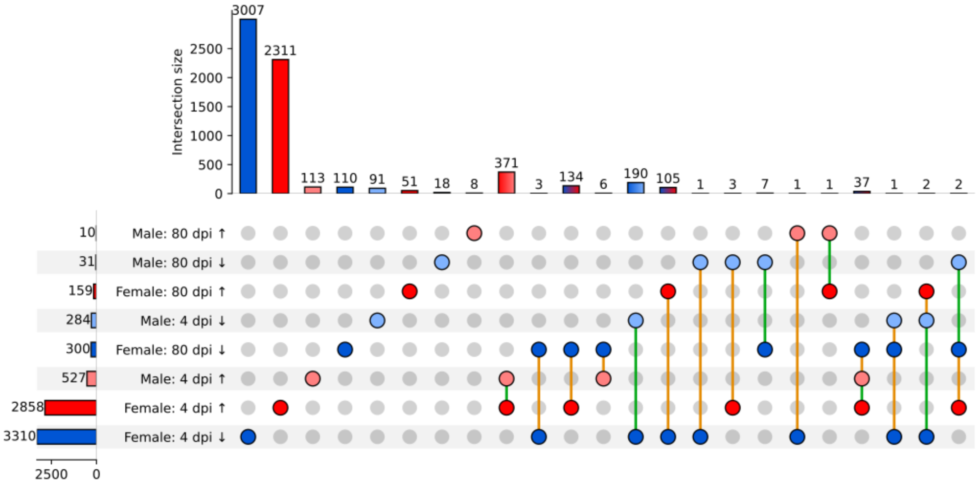

Supplementary Fig. 6. Sex- and time-dependent brainstem transcriptomic differences after intranasal SARS-CoV-2 infection in hamsters. Upset plot highlighting differences in differentially regulated gene lists grouped by sex, days post-infection, and and direction of gene regulation. Horizontal barplots show the total number of regulated genes for a comparison (separating up- and down-regulated genes). Vertical barplots show the number of genes that are specific to a comparison or shared between comparisons (according to the presence/absence of dots). Dots are colored according to the direction of regulation (red: up-regulated, blue: down-regulated) and sex (female: dark color, male: light color). Lines connecting comparisons are green when connecting comparisons from the same days post-infection and orange when connecting comparisons from different days post-infection. Related to Fig. 3-5.

Supplementary Figure 7

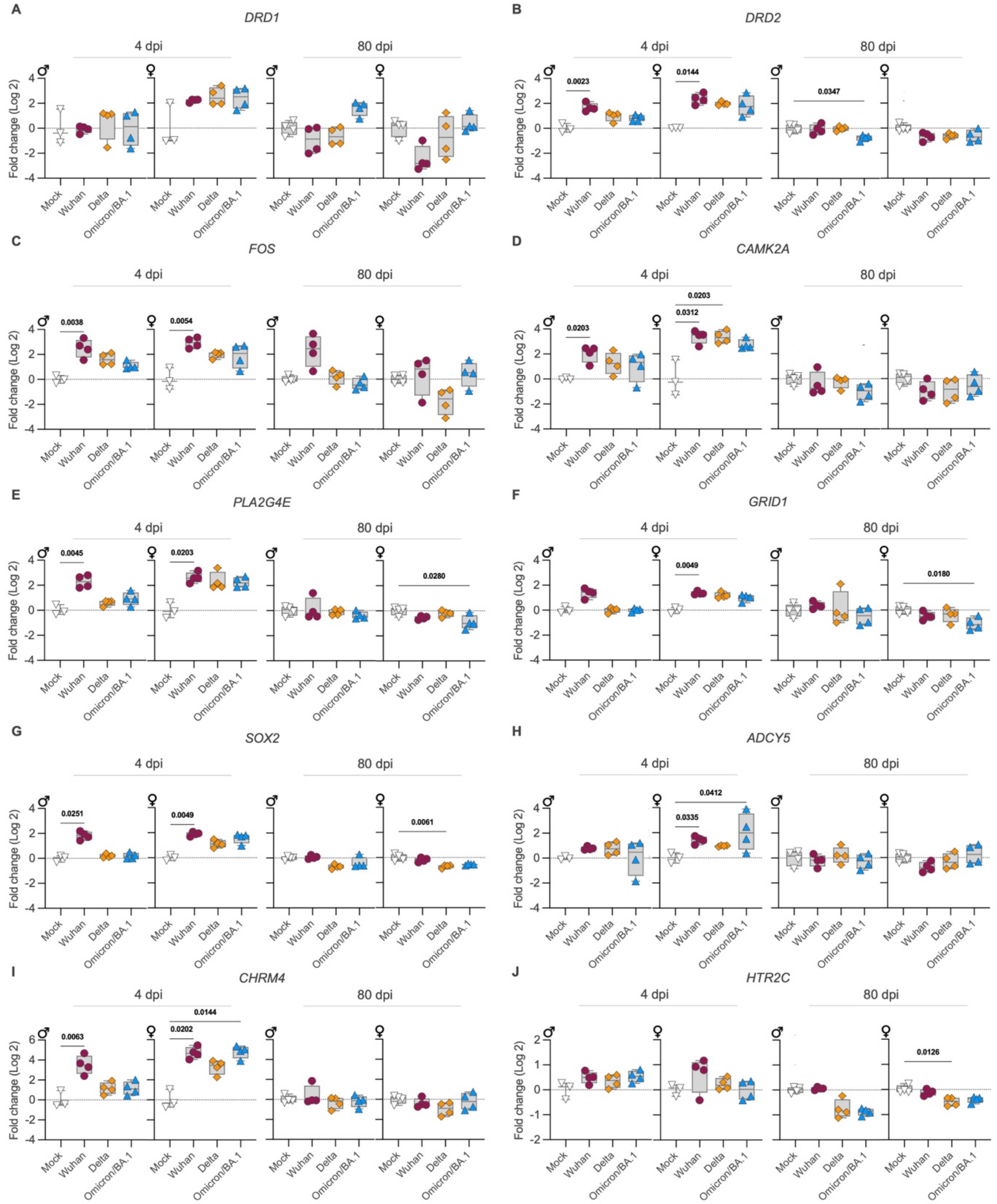

Supplementary Fig. 7. Intranasal SARS-CoV-2 infection alters the brainstem synaptic transcriptomic profile in hamsters. (A-J) Validation targets in the brainstem of male and female hamsters intranasally-inoculated with Wuhan or the variants Delta and Omicron/BA.1 at 4 and 80 dpi (n=4/group) compared to mock-infected (n=3/group at 4dpi and n=4/group at 80 dpi). Box and whisker plots (median, first and third quartiles, minimum and maximum). Individual values are also shown. Kruskal-Wallis test followed by the Dunn's multiple comparisons test (the adjusted *p* value is indicated if $p < 0.05$). Related to Fig. 4.

Supplementary Figure 8

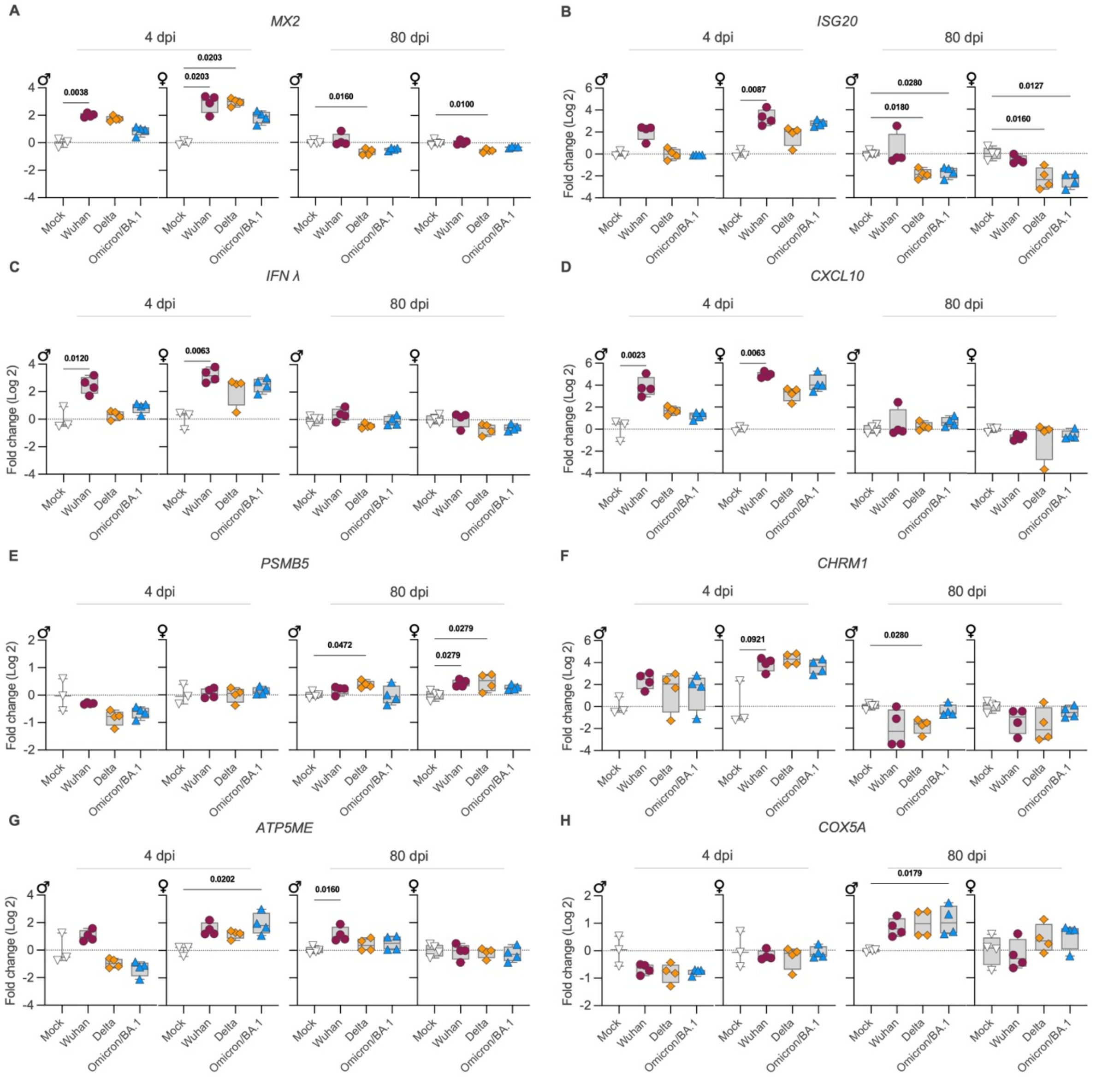

Supplementary Fig. 8. Intranasal SARS-CoV-2 infection alters the brainstem innate immune profile and induces a neurodegenerative profile in hamsters. (A-H) Validation targets in the brainstem of male and female hamsters intranasally-inoculated with Wuhan or the variants Delta and Omicron/BA.1 at 4 and 80 dpi (n=4/group) compared to mock-infected (n=3/group at 4dpi and n=4/group at 80 dpi). Box and whisker plots (median, first and third quartiles, minimum and maximum). Individual values are also shown. Kruskal-Wallis test followed by the Dunn's multiple comparisons test (the adjusted *p* value is indicated if $p < 0.05$). Related to Fig. 4,5.

Supplementary Figure 9

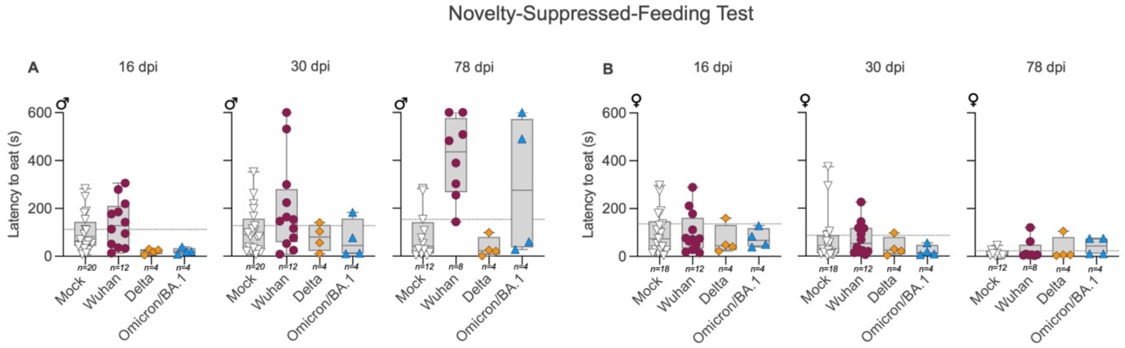

Supplementary Fig. 9. Long-term impact of SARS-CoV-2 infection on anxiety-like behavior in hamsters. (A-B) Follow-up of the novelty-suppressed feeding test assessed at 16-, 30- and 78-days post infection (dpi) in male (A) and female (B) hamsters. Increased latency to eat indicates hyponeophagia and corresponds to an anxious behavior. Box and whisker plots (median, first and third quartiles, minimum and maximum). Individual values are also shown. Horizontal dotted lines indicate the upper 95% confidence limit of the median. Related to Fig. 6.

Supplementary Figure 10

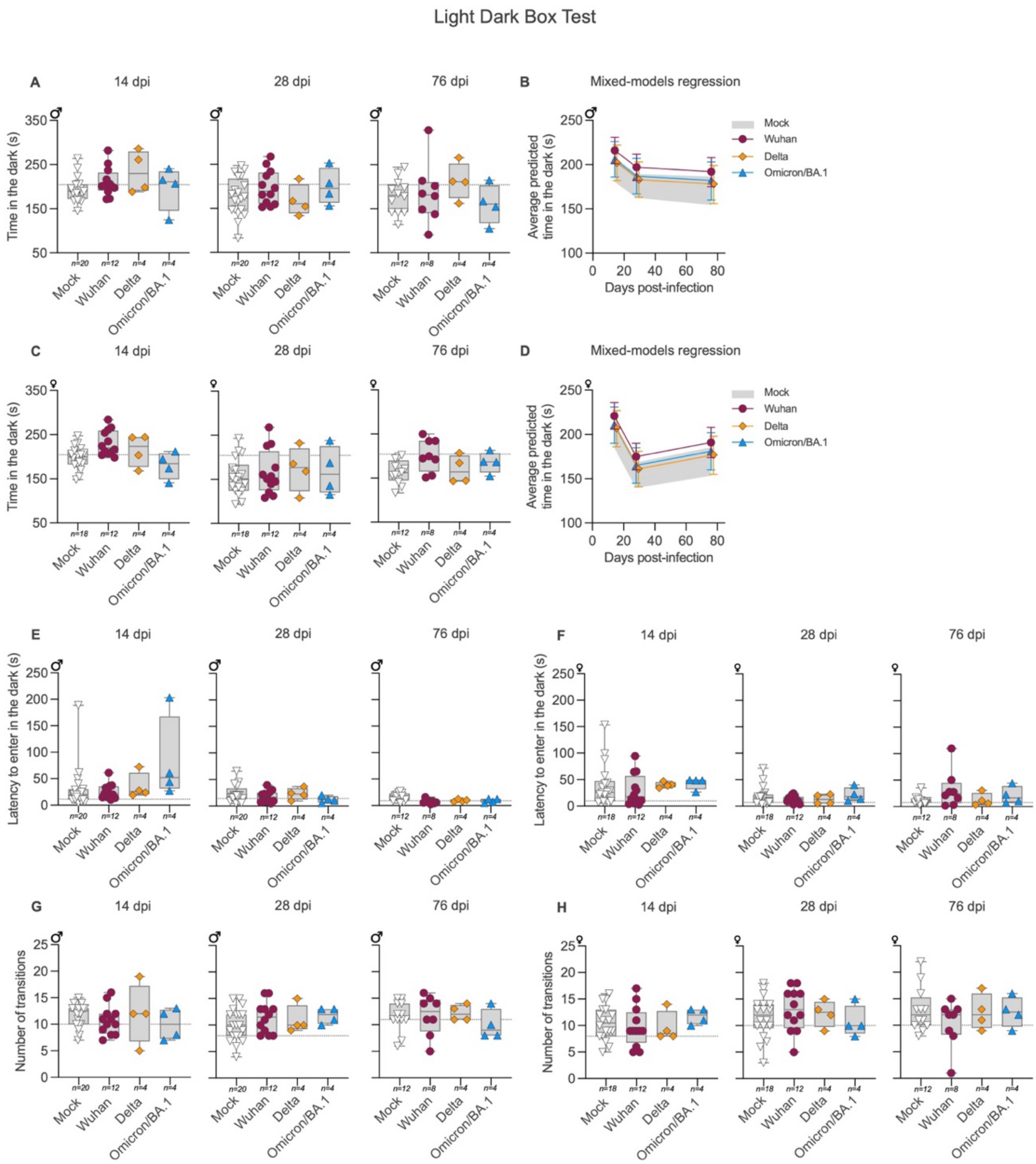

Supplementary Fig. 10. Long-term impact of SARS-CoV-2 infection on anxiety-like behavior in hamsters. (A, C) Follow-up of the light-dark box test assessed at 14-, 28- and 76-days post infection (dpi) in male (A) and female (C) hamsters. Increased time spent in the dark box corresponds to an anxious behavior. Box and whisker plots (median, first and third quartiles, minimum and maximum). Individual values are also shown. Horizontal dotted lines indicate the upper 95% confidence limit of the median. (B, D) Average predicted time in the dark analyzed by mixed-models regression in male (B) and female (D) hamsters. Horizontal lines indicate the estimated means and the 95% confidence interval. The gray crosshatched zone indicates the estimated means and the 95% confidence interval of the mock-infected group. (E-H) Secondary outputs of the test. Latency to enter in the dark chamber (E,F) and total number of transitions between the light and the dark chambers (G,H). Box and whisker plots (median, first and third quartiles, minimum and maximum). Individual values are also shown. Horizontal dotted lines indicate the upper 95% confidence limit of the median. Related to Fig. 6.

Supplementary Figure 11

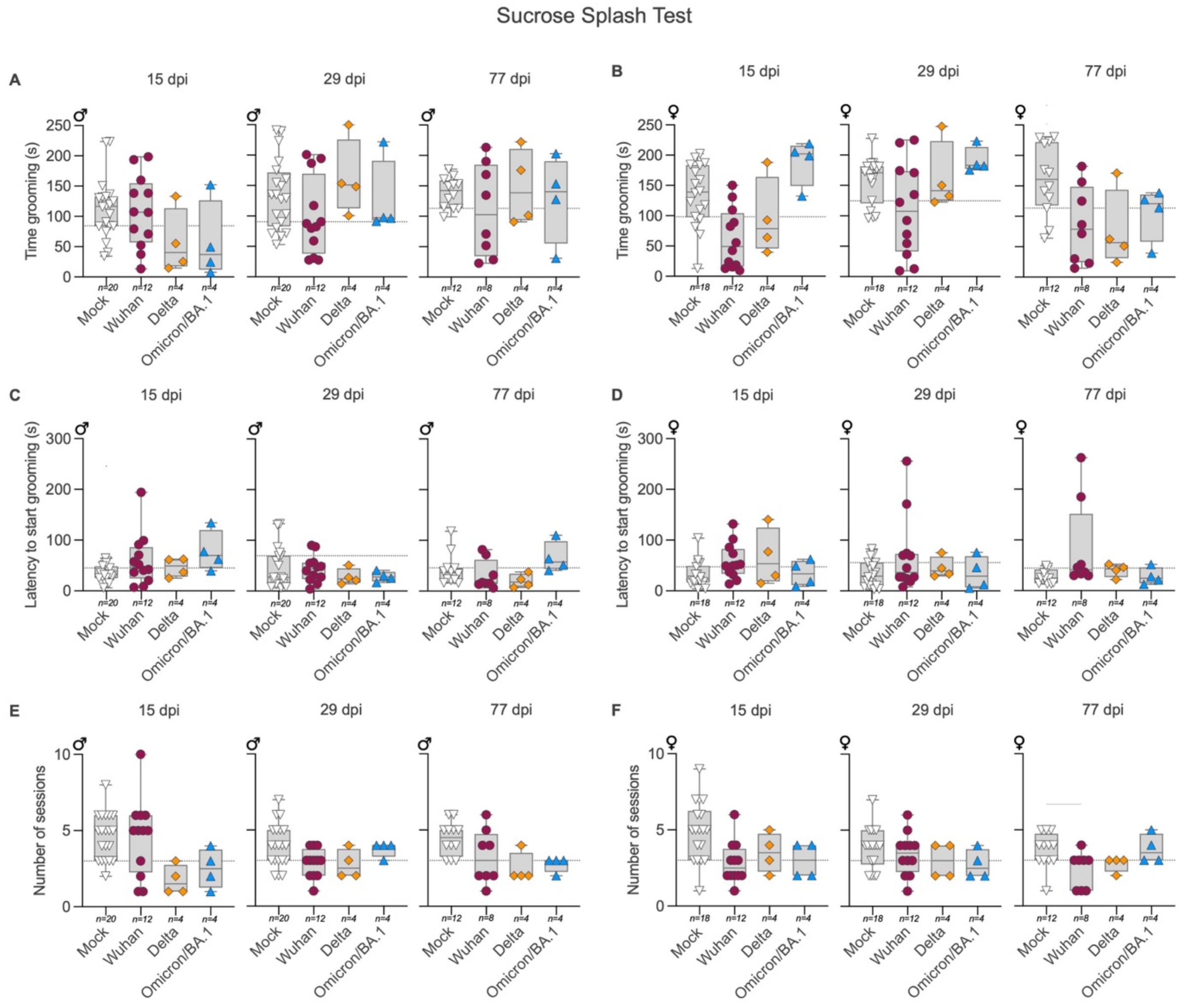

Supplementary Fig. 11. Long-term impact of SARS-CoV-2 infection on depression-like behavior in hamsters. (A-B) Follow-up of the sucrose splash test assessed at 15-, 29- and 77-days post infection (dpi) in male (A) and female (B) hamsters. Time grooming corresponds to an index of motivational and self-care behavior and decreased grooming time is considered as a depression-like behavior. (C-D) Latency to start grooming at 15, 29 and 77 days post-infection (dpi) in male (C) and female hamsters (D). (E-F) Total number of grooming sessions at 15, 29 and 77 dpi in male hamsters (E) and female (F) hamsters. Horizontal dotted lines indicate the upper (C,D) or the lower (A,B,E,F) 95% confidence limit of the median. Box and whisker plots (median, first and third quartiles, minimum and maximum). Individual values are also shown. Horizontal dotted lines indicate the lower 95% confidence limit of the median. Related to Fig. 6.

Supplementary Figure 12

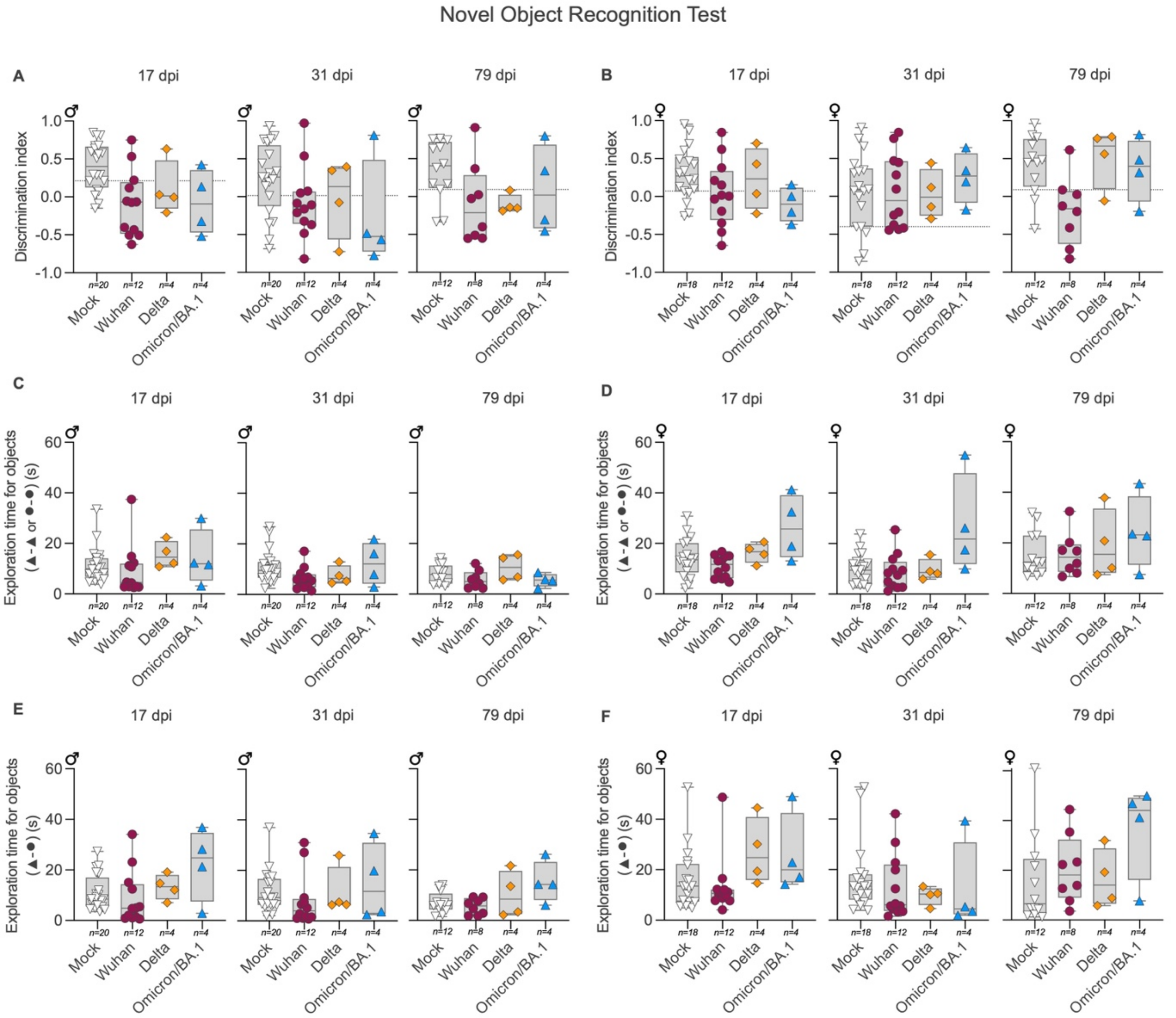

Supplementary Fig. 12. Long-term impact of SARS-CoV-2 infection on recognition memory in hamsters. (A-B) Follow-up of the novel object recognition test assessed at 17-, 31- and 79-days post infection (dpi) in male (A) and female (B) hamsters. Decreased discrimination index corresponds to less time spent exploring the new object and is indicative of short-term memory impairment. Horizontal dotted lines indicate the lower 95% confidence limit of the median. (C-D) Follow-up of exploration time for identical objects (●-● or ▲-▲) in the novel object recognition test assessed at 17-, 31- and 79- dpi in male (C) and female (D) hamsters. (E-F) Follow-up of exploration time for old and novel objects (●-▲) in the novel object recognition test assessed at 17-, 31- and 79- dpi in male (E) and female (F) hamsters. Box and whisker plots (median, first and third quartiles, minimum and maximum). Individual values are also shown. Horizontal dotted lines indicate the lower 95% confidence limit of the median. Related to Fig. 6.

Supplementary Table 1. Primer sequences used for qPCR in the golden hamster tissues

Gene symbol	Forward (5'-3')	Reverse (5'-3')	Reference
ADCY5	GGGTCCTTGGTCTCAGAAAG	GCTGTGCTCCTTGAGGTAGG	ENSMAUT00000014589.1§
ATP5ME	GCGCTAAACGCTACAGTTACC	TCGATCCGTTTCAGCTCATC	ENSMAUT00000025402.1
ACTB	GGCCAGGTCATCACCATT	GAGTTGAATGTAGTTTCGTGGATG	(Boudewijns, et al. 2020)
CAMK2A	GAAGAACGATGGTGTGAAGG	CGCACTTTGGTGTCTTCATC	ENSMAUT00000026385.1
CHRM1	ATGGTGATGCCCTTTCTCTG	GGCGTTGAGGTGTTCATTTC	ENSMAUT00000016786.1
CHRM4	GATTTCCCTGCCATAGTTGC	CAATTAGCTTCTCGGCTTGG	ENSMAUT00000009068.1
COX5A	GGGATGAACACACTTGTTGG	AACCTCCAAGATGCGAACAG	ENSMAUT00000016291.1
CXCL10	GCCATTCATCCACAGTTGACA	CATGGTGCTGACAGTGGAGTCT	(Zivcec, et al. 2011)
DRD1	AGTTTCCACGCCATAACCTG	CTGTTGCAATGTCCCACATC	ENSMAUT00000014567.1
DRD2	CTGTCATGATCGCCATTGTC	TTGTTGAGTCCGAAGAGCAG	ENSMAUT00000013955.1
FOS	AATGGTGAAGACCGTGTCAG	TCCTTTCCCTTCGGATTCTC	(de Melo, et al. 2021)
GRID1	TGGCTTCTTCCCTGGATATG	TGTCATCATTGAGGCTCAGG	(de Melo, et al. 2021)
HTR2C	ACTGGCCAGCACTTTCAATC	CACCAGCATATCAGCAATGG	ENSMAUT00000011821.1
HPRT	TGCGGATGATATCTCAACTTTAACTG	AAAGGAAAGCAAAGTTTGTATTGTCA	(Zivcec, et al. 2011)
IFNL	CCCACCAGATGCAAAGGATT	CTTGAGCAGCCACTCTTCTATG	(Boudewijns, et al. 2020)
MX2	CCAGTAATGTGGACATTGCC	CATCAACGACCTTGTCTTCAGTA	(Zivcec, et al. 2011)
PLA2G4E	CCAGGAAGAAATTGCAGTGG	CAACAGATGGCATGGAGATG	ENSMAUT00000015469.1
PSMB5	ATGCTTCATGGAACCACCAC	CTGTCTGGGAGGCAATATAAGC	ENSMAUT00000023240.1
SOX2	AGAACCCCAAGATGCACAAC	CGGTCTCCGACAAAAGTTTC	ENSMAUT00000022450.1

§ Designed by this paper, based on the Ensembl gene number, available at www.ensembl.org

References of Supplementary Table 1

Boudewijns, *et al.* STAT2 signaling restricts viral dissemination but drives severe pneumonia in SARS-CoV-2 infected hamsters. *Nat Commun* **11**, 5838 (2020).

de Melo, G. D. *et al.* Attenuation of clinical and immunological outcomes during SARS-CoV-2 infection by ivermectin. *EMBO Mol Med* **13**, e14122 (2021).

Zivcec, *et al.* Validation of assays to monitor immune responses in the Syrian golden hamster (Mesocricetus auratus). *J Immunol Methods* (2011).
