# Supplementary material for: Hamsters with long COVID present distinct transcriptomic profiles associated with neurodegenerative processes in brainstem
Source: Nat Commun. 2025 Jul 22;16:6714. doi: 10.1038/s41467-025-62048-7 (PMC12283958; doi:10.1038/s41467-025-62048-7)
Supplement: Supplementary file 2 — Reporting summary [file 41467_2025_62048_MOESM2_ESM.pdf]

nature portfolio

Corresponding author(s): Guilherme Dias de Melo

Last updated by author(s): Jul 1, 2025

Reporting Summary

Nature Portfolio wishes to improve the reproducibility of the work that we publish. This form provides structure for consistency and transparency in reporting. For further information on Nature Portfolio policies, see our Editorial Policies and the Editorial Policy Checklist.

Statistics

For all statistical analyses, confirm that the following items are present in the figure legend, table legend, main text, or Methods section.

n/a	Confirmed	
☐	☒	The exact sample size (*n*) for each experimental group/condition, given as a discrete number and unit of measurement
☐	☒	A statement on whether measurements were taken from distinct samples or whether the same sample was measured repeatedly
☐	☒	The statistical test(s) used AND whether they are one- or two-sided<br>*Only common tests should be described solely by name; describe more complex techniques in the Methods section.*
☒	☐	A description of all covariates tested
☐	☒	A description of any assumptions or corrections, such as tests of normality and adjustment for multiple comparisons
☐	☒	A full description of the statistical parameters including central tendency (e.g. means) or other basic estimates (e.g. regression coefficient) AND variation (e.g. standard deviation) or associated estimates of uncertainty (e.g. confidence intervals)
☐	☒	For null hypothesis testing, the test statistic (e.g. *F*, *t*, *r*) with confidence intervals, effect sizes, degrees of freedom and *P* value noted<br>*Give P values as exact values whenever suitable.*
☒	☐	For Bayesian analysis, information on the choice of priors and Markov chain Monte Carlo settings
☒	☐	For hierarchical and complex designs, identification of the appropriate level for tests and full reporting of outcomes
☒	☐	Estimates of effect sizes (e.g. Cohen's *d*, Pearson's *r*), indicating how they were calculated

Our web collection on statistics for biologists contains articles on many of the points above.

Software and code

Policy information about availability of computer code

Data collection	qPCR: QuantStudio 6 Flex, Applied Biosystems<br>RNAseq: Illumina NovaSeq X<br>Histology: Zen 2.6 software
Data analysis	qPCR: QuantStudio Real-time PCR System, Design and Analysis Software v. 2.7.0, Applied Biosystems<br>RNA-seq: Sequana 0.15.3<br>Histology: Zen 2.6 software and QuPath v0.5.1<br>Behavior data: R v.4.4.1 with packages lme4 (v1.1-35.3) and emmeans (v1.10.1)<br>Statistics: Prism 10 (GraphPad, version 10.2.3, San Diego)

For manuscripts utilizing custom algorithms or software that are central to the research but not yet described in published literature, software must be made available to editors and reviewers. We strongly encourage code deposition in a community repository (e.g. GitHub). See the Nature Portfolio guidelines for submitting code & software for further information.

Data

Policy information about availability of data

All manuscripts must include a data availability statement. This statement should provide the following information, where applicable:

- Accession codes, unique identifiers, or web links for publicly available datasets
- A description of any restrictions on data availability
- For clinical datasets or third party data, please ensure that the statement adheres to our policy

RNA preparation was used to build libraries using an Illumina Stranded mRNA library Preparation Kit (Illumina, USA) following the manufacturer's protocol. Quality control was performed on an Agilent BioAnalyzer. Illumina NextSeq2000 was used to produced single-end 75b reads for the 4 dpi libraries (accession: E-MTAB-14779) and Illumina NovaSeq X sequencer was used to produce paired-end 150b reads for 80 dpi RNA-seq libraries (accession: E-MTAB-14780). All other data generated or analyzed du ring this study are included in this article along with its extended and supplementary data.

Data availability
All data associated with this study are present in the paper or the Supplementary Materials. Source data are provided with this paper.
The RNA-seq data are available in the Array Express database (https://www.ebi.ac.uk/biostudies/arrayexpress): 4dpi: ArrayExpress E-MTAB-14779; 80 dpi: ArrayExpress E-MTAB-14780.
The mixed-effect model analyses for behavioral tests are available in the Git repository: https://gitlab.pasteur.fr/hub/19421-long-covid-brainstem.

Research involving human participants, their data, or biological material

Policy information about studies with human participants or human data. See also policy information about sex, gender (identity/presentation), and sexual orientation and race, ethnicity and racism.

Reporting on sex and gender	n/a
Reporting on race, ethnicity, or other socially relevant groupings	n/a
Population characteristics	n/a
Recruitment	n/a
Ethics oversight	n/a

Note that full information on the approval of the study protocol must also be provided in the manuscript.

Field-specific reporting

Please select the one below that is the best fit for your research. If you are not sure, read the appropriate sections before making your selection.

☒ Life sciences ☐ Behavioural & social sciences ☐ Ecological, evolutionary & environmental sciences

For a reference copy of the document with all sections, see nature.com/documents/nr-reporting-summary-flat.pdf

Life sciences study design

All studies must disclose on these points even when the disclosure is negative.

Sample size	We use the minimum number of animais necessary to achieve statistical representativity (n=8 for behavioral tests / n=4 for molecular tests) based on previous power analysis and data observed using this model.
Data exclusions	No data were excluded.
Replication	Behavioral data come from four independent experiments.
Randomization	There was no randomization due to pre-defined housing conditions (separeted isolators between infected and non-infeted animais).
Blinding	Blinding was not possible du ring in vivo analysis due to pre-defined housing conditions (separeted isolators between infected and non-infeted animais). Ex vivo analysis were blinded (coded samples).

Reporting for specific materials, systems and methods

We require information from authors about some types of materials, experimental systems and methods used in many studies. Here, indicate whether each material, system or method listed is relevant to your study. If you are not sure if a list item applies to your research, read the appropriate section before selecting a response.

Materials & experimental systems

n/a	Involved in the study
☐	☒ Antibodies
☐	☒ Eukaryotic cell lines
☒	☐ Palaeontology and archaeology
☐	☒ Animals and other organisms
☒	☐ Clinical data
☒	☐ Dual use research of concern
☒	☐ Plants

Methods

n/a	Involved in the study
☒	☐ ChIP-seq
☒	☐ Flow cytometry
☒	☐ MRI-based neuroimaging

Antibodies

Antibodies used	Mouse anti-SARS-CoV-2 nucleocapsid (MA-29981, Invitrogen)<br>Rabbit anti-SARS-CoV-2 spike (GTX135356, GeneTex)<br>Rabbit anti-Iba-1 (019-19741, Wako chemical)<br>Chicken anti-GFAP (ab4674, Abcam)
Validation	Commercial validated antibodies

Eukaryotic cell lines

Policy information about cell lines and Sex and Gender in Research

Cell line source(s)	Vero E6 (ATCC #CRL-1586)
Authentication	Cell lines were not authenticated.
Mycoplasma contamination	Cell lines were not tested
Commonly misidentified lines (See ICLAC register)	No commonly misidentified cell lines were used in the study

Animals and other research organisms

Policy information about studies involving animals; ARRIVE guidelines recommended for reporting animal research, and Sex and Gender in Research

Laboratory animals	golden hamsters (Mesocricetus auratus; RjHan:AURA) of 5-6 weeks of age (average weight 60-80 grams)
Wild animals	n/a
Reporting on sex	both male and female animals were used
Field-collected samples	n/a
Ethics oversight	All animal experiments were performed according to the French legislation and in compliance with the European Communities Council Directives (2010/63/UE, French Law 2013–118, February 6, 2013) and according to the regulations of Pasteur Institute Animal Care Committees. The Animal Experimentation Ethics Committee (CETEA 89) of the Institut Pasteur approved this study (200023; APAFIS#25326-2020050617114340 v2) before experiments were initiated.

Note that full information on the approval of the study protocol must also be provided in the manuscript.

Plants

Seed stocks	*Report on the source of all seed stocks or other plant material used. If applicable, state the seed stock centre and catalogue number. If plant specimens were collected from the field, describe the collection location, date and sampling procedures.*
Novel plant genotypes	*Describe the methods by which all novel plant genotypes were produced. This includes those generated by transgenic approaches, gene editing, chemical/radiation-based mutagenesis and hybridization. For transgenic lines, describe the transformation method, the number of independent lines analyzed and the generation upon which experiments were performed. For gene-edited lines, describe the editor used, the endogenous sequence targeted for editing, the targeting guide RNA sequence (if applicable) and how the editor was applied.*
Authentication	*Describe any authentication procedures for each seed stock used or novel genotype generated. Describe any experiments used to assess the effect of a mutation and, where applicable, how potential secondary effects (e.g. second site T-DNA insertions, mosiacism, off-target gene editing) were examined.*
